# Predicting the spread-risk potential of chronic wasting disease to sympatric ungulate species

**DOI:** 10.1080/19336896.2020.1720486

**Published:** 2020-02-03

**Authors:** Catherine I. Cullingham, Rhiannon M. Peery, Anh Dao, Debbie I. McKenzie, David W. Coltman

**Affiliations:** a Department of Biological Sciences, University of Alberta, Edmonton, Canada; b Department of Biology, Carleton University, Ottawa, Canada

**Keywords:** Chronic wasting disease, Prnp, wildlife disease, phylogenetics, species transmission barriers

## Abstract

Wildlife disease incidence is increasing, resulting in negative impacts on the economy, biodiversity, and potentially human health. Chronic wasting disease (CWD) is a fatal, transmissible spongiform encephalopathy of cervids (wild and captive) which continues to spread geographically resulting in exposure to potential new host species. The disease agent (PrP^CWD^) is a misfolded conformer of the cellular prion protein (PrP^C^). In Canada, the disease is endemic in Alberta and Saskatchewan, affecting mule and white-tail deer, with lesser impact on elk and moose. As the disease continues to expand, additional wild ungulate species including bison, bighorn sheep, mountain goat, and pronghorn antelope may be exposed. To better understand the species-barrier, we reviewed the current literature on taxa naturally or experimentally exposed to CWD to identify susceptible and resistant species. We created a phylogeny of these taxa using cytochrome B and found that CWD susceptibility followed the species phylogeny. Using this phylogeny we estimated the probability of CWD susceptibility for wild ungulate species. We then compared PrP^C^ amino acid polymorphisms among these species to identify which sites segregated between susceptible and resistant species. We identified sites that were significantly associated with susceptibility, but they were not fully discriminating. Finally, we sequenced Prnp from 578 wild ungulates to further evaluate their potential susceptibility. Together, these data suggest the host-range for CWD will potentially include pronghorn, mountain goat and bighorn sheep, but bison are likely to be more resistant. These findings highlight the need for monitoring potentially susceptible species as CWD continues to expand.

## Introduction

There is an increasing incidence of wildlife disease globally [1–3], and the impacts of these diseases have cascading effects. These range from affecting ecosystem health [4], to spillover to wildlife and humans [2,5], to the economic burden of controlling and/or managing the disease [6]. Chronic wasting disease (CWD), a fatal prion disease of cervids, is considered an emerging threat to biodiversity because of its impacts on the host species and the potential long-term effects on ecosystem function [7].

Chronic wasting disease is a particularly challenging wildlife disease to manage as it is transmitted both directly (individual to individual), or indirectly (contaminated environment to individual) [8,9]. First documented in the 1960s in Colorado in captive mule deer (*Odocoileus hemionus*) [10], it has spread into wild cervid populations, and continues to increase in prevalence, and geographic range (United States, Canada, South Korea, Norway, Sweden, Finland) [11,12]. The host range has also expanded with CWD infections occurring in white-tail deer (*Odocoileus virginianus*), elk (*Cervus canadensis*), moose (*Alces alces*), sika deer (*Cervus nippon*), red deer (*Cervus elaphus*), and reindeer (*Rangifer tarandus*) [13–15]. Continued host-range expansion is a major concern. In Canada, the disease currently affects mule deer, white-tail deer, moose, and elk; these species are sympatric with other species (caribou, bighorn sheep (*Ovis canadensis*), mountain goat (*Oreamnos americanus*), bison (*Bison bison*), and pronghorn (*Antilocapra americana*)) that could be at risk. If we can better understand species susceptibility, we may be able to manage spread to novel hosts and reduce negative impacts.

The CWD disease agent is a misfolded, aggregated version (PrP^CWD^) of the cellular prion protein (PrP^C^), and the success of disease transmission has been linked to the similarity of the amino acid sequence of PrP^CWD^ to the cellular host protein, PrP^C^ [16,17]. Kurt & Sigurdson [18] used CWD transmission data across studies of model and non-model organisms to understand whether there were regions of PrP^C^ that were important in determining disease susceptibility. While they found an association of successful conversion when the β2-α2 loop of the protein of the host was similar to the infectious agent, they concluded it was not the only region critical for successful prion replication.

**Figure 1.**
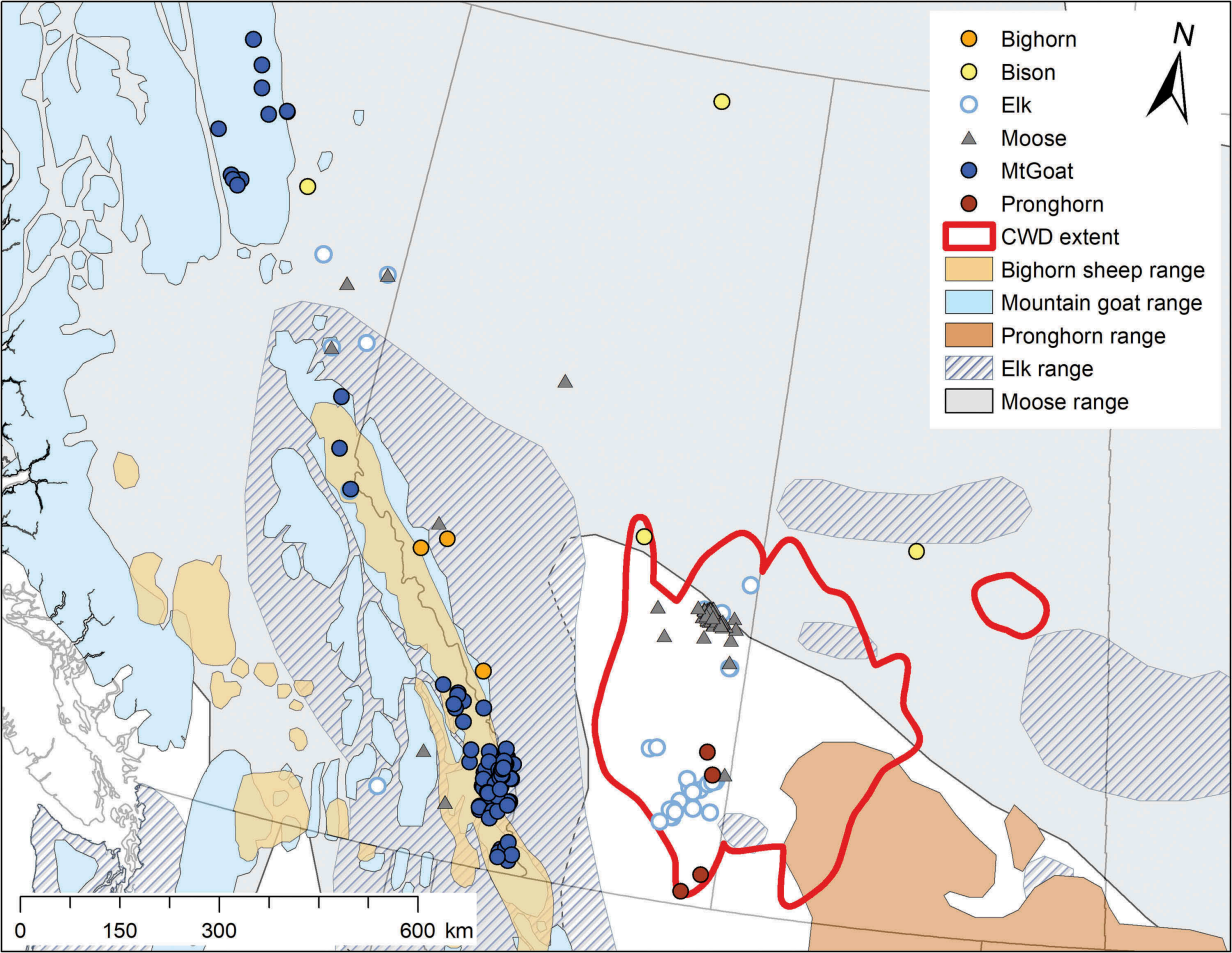
Distribution of sympatric species samples, and ranges, in relation to an approximation of the extent of CWD in wild populations in Alberta and Saskatchewan (based on incidence data up to 2016). Species ranges were obtained from the IUCN Red List of Threatened Species (https://www.iucnredlist.org/), these ranges are approximate.

Given that the similarity between host PrP^C^ and the infectious prion is an important factor for successful disease transmission, we hypothesize that the evolutionary relatedness of species may predict their susceptibility to CWD. To test this, we built a phylogeny using species with known CWD susceptibility using cytochrome B (cytB) sequences. We then used this phylogeny to test if CWD susceptibility is explained by the relatedness among species e.g. [19,20]. The methodology we used requires a quantitative trait – susceptible or resistant to prion infection. While the true distribution of this trait is presumably quantitative across a continuum, it is has largely been unobserved, therefore we have chosen to use a binary definition. We defined a species as resistant if oral challenge and/or intracerebral inoculation did not result in clinical disease, and susceptible if oral challenge or natural infection resulted in disease. To be conservative, we did not include species that could be infected by routes other than the oral route [21–23] as these species would not likely be susceptible in a natural setting [18]. We then added ungulate species with unknown susceptibility to CWD infection from within and adjacent to the CWD endemic region in Canada (Figure 1) to the phylogeny to estimate their probability of CWD susceptibility. Next, we examined the amino acid sequences of susceptible, and resistant species to identify polymorphisms associated with susceptibility to identify protein regions involved in the conversion process. Finally we sequenced the *Prnp* coding region in bison, bighorn sheep, elk, moose, mountain goats, and pronghorn from within and adjacent to the CWD endemic region in Canada (Figure 1) to further evaluate their potential susceptibility.

## Results

### Phylogenetic analysis

We aligned cytB sequences from 20 species (27 haplotypes; Table S1), for 277 characters, of which 64 were variable. The maximum likelihood estimate of phylogenetic relationships for cytB did not disagree with accepted mammalian phylogenetic relationships (Figure S1). The subset of taxa with susceptibility information was used to determine the correlation between resistance/susceptibility to CWD and phylogenetic signal. We found that, with and without branch lengths as a factor, CWD resistance/susceptibility is highly correlated (*p* ≤ 0.003) with phylogenetic history (Figure S2).

**Figure 2.**
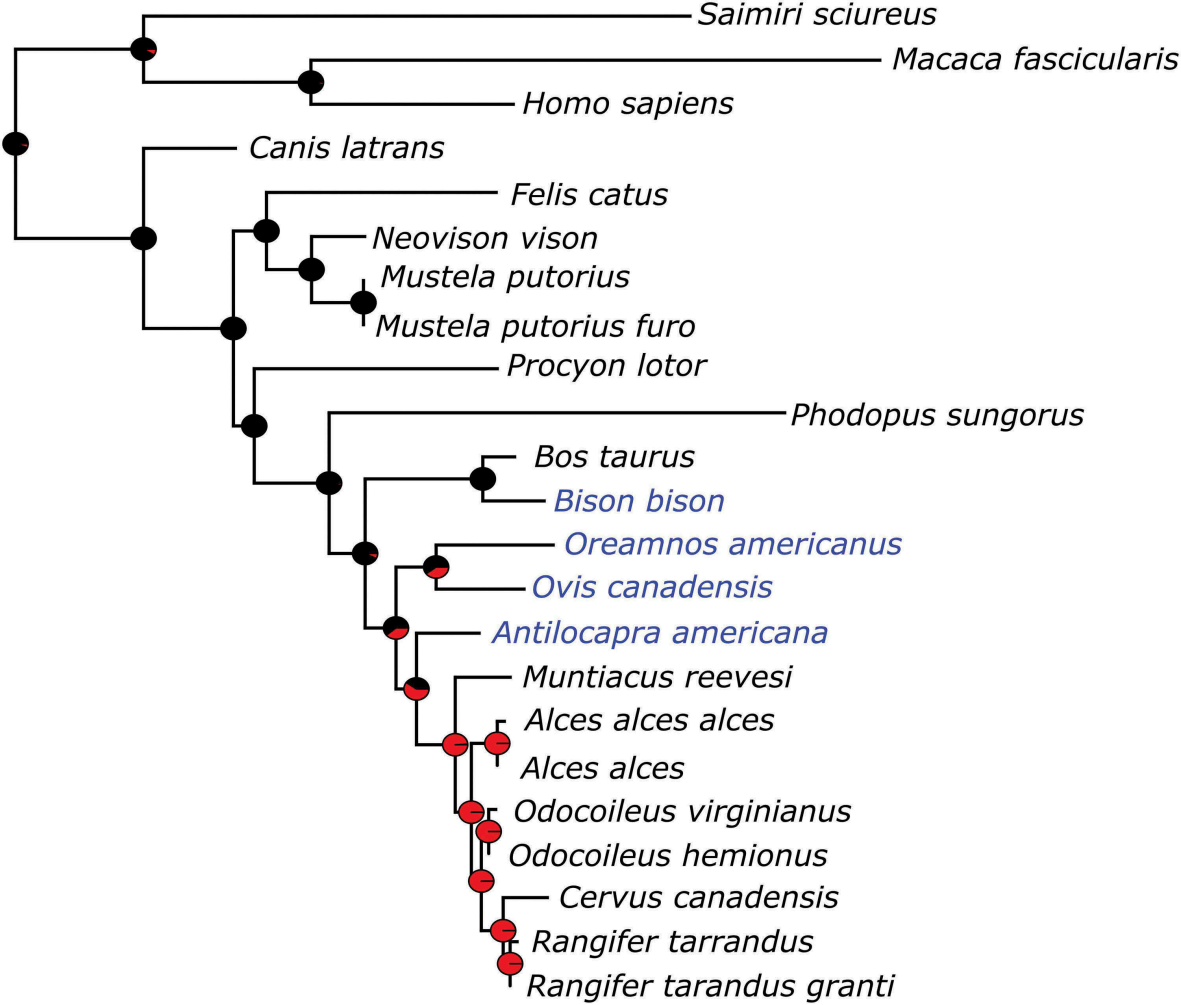
Maximum likelihood phylogram of cytochrome b genes with taxa for species with known (species names in black) and unknown (species names in blue) CWD susceptibility. Pie charts at nodes indicate predicted ancestral resistance (black colouring) or susceptibility (red colouring).

Inclusion of the species with unknown susceptibility in the phylogeny allowed us to predict the probability of CWD susceptibility. From this, bison are predicted to be resistant, while pronghorn, bighorn sheep, and mountain goat are likely to be susceptible, with pronghorn more likely to be susceptible than bighorn sheep and mountain goat (Figure 2).

### Identifying CWD associated polymorphisms

**Figure 3.**
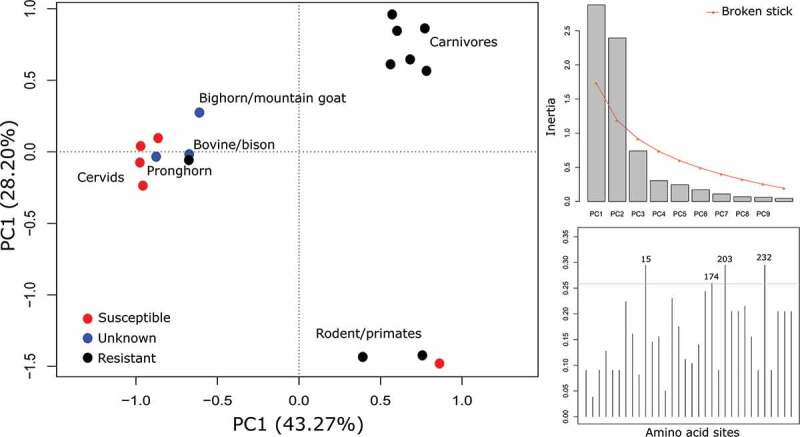
Principle component analysis of amino acids sites that are variable between species that are resistant and susceptible to CWD, with unknowns included. The plot on the top right is a scree plot showing the first two axes are significant and explain most of the variation in the data. The bottom right plot is the loadings of the amino acid sites on the first axis, the top 10% are labelled. The scree plot and loadings plot are based on only the resistant and susceptible data (biplot not shown).

We aligned 26 unique PrP amino acid sequences from 14 species to human PrP; sequences ranged from 253 a.a. to 272 a.a in length. Removing the invariant sites reduced the matrix to 30 sites and 15 haplotypes (9 ‘resistant’, and 6 ‘susceptible’). From the Fisher’s exact test, 11 sites significantly segregated susceptible and resistance haplotypes (Table 3). The majority of the variation in the sequence matrix (30 sites) was explained by the first two components of the PCA (74.52%). There was a clear distinction between susceptible and resistant species along the first axis, and five of the 11 sites identified from the Fisher’s exact test were in the top 10% of loadings for that axis (Figure 3; Table 3). The second axis separated the carnivores from rodents and primates. The unknown sequences were added to the PCA analysis to determine whether they are placed similarly to the phylogenetic tree (Figure 3); pronghorn are clustered with cervids, while bison and bighorn sheep/mountain goat are placed close to the cervid cluster.

**Table 1.** Species that have been orally exposed to CWD agent, either experimentally or in natural populations. Species that are susceptible ‘S’, are species found positive following oral exposure to CWD prions, while resistant species ‘R’ are those who are resistant following intracerebral inoculation, or oral exposure to CWD prions. We have included the accession number(s) for their PrP amino acid sequence, and the reference(s) for their susceptibility status.

Species	CWD susceptibility	Accession/ Reference	Reference
Caribou	S	AAZ81476.1	[14,72]
		AAZ81475.1	
		AAZ81473.1	
		AFF27616.1	
		AFF27615.1	
Cat	R	XP_019682354.2	[22]
		AGA63675.1	
Cow	R	NP_001258555.1	[26]
		AAV30481.1	
		AAV30479.1	
		AAV30478.1	
Coyote	R	AGA63673.1	[24]
		AGA63672.1	
Djungarian hamster	R	ACG63356.1	[73]
Elk	S	ABW79908.1	[74,75]
		ABW79904.1	
Ferret	R	XP_004772889.1	[21]
Macaques	R	BAD51981.1	[25,49]
Mink	R	P40244	[23]
Moose	S	AFF27617.1	
		AAT77255.1	
Mule deer	S	AAR01535.1	
Muntjack deer	S	BAO18775.1	[76]
Raccoon	R	ACA50738.1	[77]
Squirrel monkey	S	AET34447.1	[25]
White-tail deer	S	AAP37447.1	
		XP_020739306.1	
		[78]

**Table 3.** PrP reduced sequence matrix describing the eleven sites that are significantly different between susceptible (S), and resistant (R) species to CWD oral inoculation, site numbers refer to human PrP. Amino acid sites that were in the top 10% of loadings in the principle component analysis are highlighted in grey. Species/sequences that have not been exposed to CWD orally are indicated as of unknown status (U). Deletions are indicated by “-“.

Amino acid site	15	97	170	174	203	205	215	232	237	246	249
Significance value (*p*)	0.011	0.011	0.011	0.002	0.011	0.017	0.017	0.011	0.017	0.017	0.017
Djungarian hamster (R)	A	N	N	N	V	M	I	A	S	F	F
Bovine, Bison01 (R)	M	G	S	N	I	M	I	-	S	F	F
Cat, ferret, mink, coyote, raccoon (R)	T	G	S	N	M	I	V	A	P	L	L
Coyote (R)	T	S	S	N	M	I	V	A	P	L	L
Squirrel monkey (S) & macaques (R)	T	N	S	N	V	M	I	M	S	F	F
Cervids (S)	M	S	N	T	I	M	I	-	S	F	F
Human (R)	T	S	S	N	V	M	I	M	S	F	F
Moose100R (U)	M	R	N	T	I	M	I	-	S	F	F
Bighorn, mountain goat (U)	M	S	S	N	I	I	I	-	S	F	F
Bison02 (U)	T	G	S	N	I	M	I	-	S	F	F
Pronghorn (U)	M	S	S	T	I	M	I	-	S	F	F

### Sequence assembly and polymorphism analysis

Following assembly and alignment, there were very few PrP amino acid polymorphisms within each species. Among the 259 bighorn sheep and mountain goats sequenced, there was only one haplotype identified. This haplotype is identical to the ARQ haplotype in domestic sheep (NP_001009481). Among bison sampled, two haplotypes were identified, one identical to bovine (NP_001258555), and the other to a previously characterized bison PrP (AAV30502). All sampled elk carried the 226E polymorphism (P67986), while a small number were heterozygous with the 132L polymorphism (AAF80281). Moose were the most variable, with three haplotypes identified: the cervid wildtype (AAT77255), the 209I polymorphism (AA067544), and a previously undescribed haplotype which has the 209I polymorphism and an amino acid change from serine (S) to arginine (R) at site 100 (submitted to Genbank: MN970212). This polymorphism was found in one heterozygous individual and was Sanger sequenced to confirm the mutation. These above amino acid numberings refer to cervid PrP. The amino acid differences between the at-risk haplotypes, and the susceptible and resistant haplotypes at the 11 sites associated with CWD susceptibility are presented in Table 3.

## Discussion

Through reviewing the current literature on CWD oral exposure data and trait association analyses, we determined that CWD susceptibility is significantly correlated with species phylogeny. We used this information to predict the potential susceptibility of ungulate species sympatric with mule deer and white-tail deer in Alberta, British Columbia, and Saskatchewan. Our data suggests that bison are resistant, while pronghorn, bighorn sheep, and mountain goat are potentially susceptible. In addition, we identified amino acid sites that discriminate between resistant and susceptible haplotypes, this information will be important in building our understanding of what PrP regions play a role in misfolding and species transmission barriers. Finally, we characterized the PrP sequence for bighorn sheep, bison, elk, moose, mountain goat, and pronghorn within or near the Canadian CWD endemic zone as a resource for understanding regional susceptibility.

Given the importance of the compatibility between PrP^C^ and PrP^CWD^ for successful disease transmission, the strong phylogenetic signal found for disease susceptibility is expected. This signal allows us to assess the potential spread risk to sympatric ungulates in the Canadian CWD enzootic area. Our analysis predicts that pronghorn antelope are more likely to be susceptible to CWD than any of the species we tested; bighorn sheep and mountain goat have a small probability of susceptibility, while bison are predicted to be resistant. We recognize that there are potential issues with our definition of resistant and susceptible species. Oral dosing varied among studies, both in the volume of dose [21,22], method of dosing [24,25], but also in the course (e.g. one day [24,26], multiple days [23,25]). We view infectibility as a probability along a spectrum of susceptibility which correlates well with literature on studies that have used intracerebral inoculations. For example, Prnp sequence for mountain goat and big horn sheep is the same as sheep, and Suffolk sheep are susceptible to CWD following intracerebral inoculation [27]. Cattle, which share prion sequence with bison, are also susceptible to CWD following intracerebral inoculation [28]. Therefore, the potential resistance of these species will likely depend on multiple factors including CWD strain, host genotype, and infectious dose. As the disease continues to spread, using phylogenetics could prove useful in helping to target management resources to those species that are most likely to be susceptible to the disease. Our current analysis highlights pronghorn antelope should be prioritized for inclusion in CWD surveillance activities given the proximity of the enzootic region in Canada to the pronghorn distribution.

While our samples are from Canadian populations of these potentially at-risk species, the data likely represent the spread risk for many in North America. The PrP region has been sequenced in bighorn sheep in the United States from populations in Washington [29], and they found the single haplotype we identified. Similarly for bison, Seabury et al. [30] sequenced individuals in four state parks, and found the same genetic variants we identified in the Canadian populations. Also, if there are other variants that we have not identified, they are likely very similar to the ones we have identified and would place similarly in the phylogeny.

Previous studies examining species barriers to CWD transmission have identified the similarity of the β2-α2 loop region as an important determinant for susceptibility [31–33]. In particular, the amino acid sites 170 and 174 are key to supporting transmission [34,35]. Our identification of significant association with resistance at these sites (Table 3) fits well with these previous findings. However, it has also been noted that they are not the only sites important in determining the species barrier [36,37]. Our identification of additional sites may help to better understand their impact on maintaining the stability of the protein. For example, three sites we identified (203, 215, 232), also contribute to human prion disease reviewed in [38]. More recently, site 205 (208 in deer) was identified by Harrathi *et al*. [39] as a key site for conversion of PrP^CWD^ in sheep. However, Priola & Chesebro [40] identified amino acid 138 as important in preventing hamsters from converting mouse scrapie, which indicates important sites may differ among the transmissible encephalopathies.

Metal and protein binding is thought to promote folding and stabilize folded proteins [41,42]. Some experimental data suggest altered metal homoeostasis may be involved in the development of TSE diseases, where amino acid residues in the disordered region of the PrP protein may play a role in the interaction of PrP^CWD^ with PrP^C^ (reviewed in [41]). Residue changes in this region may, therefore, affect the metal binding ability, and result in a less stable PrP^C^ that is more prone to misfolding. We identified site 97 as significantly different between susceptible and resistant haplotypes and this site is in the disordered region of the PrP protein, adjacent to one of the Cu^2+^ binding sites: histidine 96 [43]. This may alter the disease response of an individual within a species, for example, white-tail deer with mutations in this disordered region (e.g. 95H (hu91) and 96S (hu92)) are underrepresented in the diseased population [44,45]. While it does not confer resistance, it does suggest this region likely plays an important role in disease modulation. Finally, there were a number of sites identified in the C-terminal region, which potentially interacts with the Cu^2+^ binding region [46] which may also alter metal binding homoeostasis and promote misfolding.

Squirrel monkeys are orally susceptible to CWD [25,47], however, the time to disease onset is considerably longer in comparison to other TSE diseases [48]. This species is not proximate to susceptible species in the phylogeny (Figure 2), they do not carry the same polymorphisms as cervids at many of the susceptible associated sites (Table 3), but rather have the same haplotype at the 11 CWD segregating sites as macaques, which are currently considered resistant to CWD [49]. One difference between the squirrel monkey and macaque PrP is an extra octapeptide repeat, which is also variable within squirrel monkeys. In the CWD oral inoculation study conducted by Race et al. [25,47], there were three squirrel monkeys heterozygous for the number of repeats (four and five), the remaining individuals were homozygous for the extra repeat; the heterozygous individuals had extended incubation periods [47]. It is plausible that the additional fifth repeat makes this PrP more unstable and, therefore, prone to misfolding. Similarly, Goldfarb et al [50]. found that humans with higher numbers of the octapeptide repeat were predisposed to Creutzfeldt-Jakob disease.

By comparing polymorphisms in PrP^C^ between orally susceptible and resistant species to CWD, we have identified 11 amino acid sites, five of which are newly identified, that may contribute to the species transmission barrier. We are not suggesting these are the only factors that will determine the species barrier, the species barrier is also affected by CWD strain [36], PrP^CWD^ structure and its interaction with the native PrP^C^ [51]. Instead this information may be useful in prioritizing experimental studies to help identify other species that may be susceptible to CWD. For example, our approach can identify candidate species for developing transgenic mice for testing transmission potential [52,53]. Knowledge of susceptibility-associated sites may lead to a better understanding of the interactions involved in the conversion of PrP^C^ to PrP^CWD^. Most importantly, these data allow us to prioritize management and monitoring of natural populations to target species with a greater probability of CWD infection as CWD prevalence and geographic range continue to expand.

## Methods

### Phylogenetic analysis

Based on approximate mutation rate and phylogenetic agreement with published mammalian phylogenies [54] we chose cytB to estimate the phylogeny upon which we tested whether susceptibility to CWD can be explained by phylogenetic history. We obtained cytB amino acid sequences from GenBank for eight species resistant to CWD following oral inoculation (bovine (*Bos taurus*), macaques (*Macaca fascicularis*), ferret (*Mustela putorius furo*), cat (*Felis catus*), mink (*Neovison vison*), and coyote (*Canis latrans*)), or intracerebral exposure (raccoon (*Procyon lotor*), and Djungarian hamster (*Phodopus sungorus*)), and seven species known to be susceptible following natural exposure or oral inoculation (squirrel monkey (*Saimiri sciureus*), mule deer, white-tail deer, elk, muntjack deer (*Muntiacus reevesi*), moose, and caribou). We included human (*Homo sapiens*) as an outgroup and for numbering reference. For details on sequence accessions, and references to susceptibility studies, see Table 1. Cytochrome B amino acid sequences were aligned with the MAFFT v7.419 plugin within Mesquite [55]. Phylogenetic relationships were estimated in RAxML v8.2.10 [56] on the CIPRES Science Gateway [57] with the following parameters: exclude characters 2–6, find best tree, automatically stop bootstrap replicates, and use the MTMAM model (Supplementary Information). Susceptibility was mapped onto the phylogeny using the parsimony method for categorical data within Mesquite. To determine if susceptibility is significantly correlated with phylogenetic relationship we used the autocorrelation test Abouheif’s *C_mean_* [58], which tests for independence of trait values, with the *abouheif.moran* command and 999 permutations within the R package adephylo v1.1–11 [59]. This test requires a complete matrix for the character being tested, therefore human was assumed to be resistant in this analysis, currently there is no data to suggest that humans are susceptible to CWD [60]. To estimate ancestral states and predict susceptibility to CWD we added wild ungulate species at risk (bighorn sheep, bison, mountain goat, pronghorn) to our cytB phylogeny. We tested the phylogeny using the equal rates, maximum likelihood ancestral character estimator within the R packages ape v5.3 [61] and phytools v0.6–99 for tree drawing [62].

### Identifying CWD associated polymorphisms

Amino acid sequence data for the PrP protein of all species used in the phylogeny were obtained from GenBank, and these were aligned using ClustalX [63], and refined using the Opal plugin [64] with default parameters in Mesquite v3.51 build 898 (Maddison & Maddison, 2018) and verified by eye. Using this alignment, we removed all invariant sites, where we considered a site invariant if ≤ 2 sequences were different. We then collapsed haplotypes into unique sequences and recoded the amino acid to 0 and 1; 1 if the amino acid was the same as the cervid wildtype, and 0 if not. For the remaining haplotypes, we completed Fisher’s exact tests on the polymorphic sites to determine whether any of the polymorphisms were associated with CWD susceptibility. Some of the sites may be linked, and will not represent independent sites therefore, we analysed the matrix of variable sites using principle component analysis (PCA) to validate our findings. We used the R package *vegan* [65] to do the PCA, and *adegenet* [66] to produce a plot of the loadings on the first axis of the principle components to identify the amino acid sites discriminating the resistant and susceptible species. Finally, we applied these same methods to bighorn sheep, bison, mountain goat, and pronghorn to determine if amino acid state can be used to predict susceptibility in the unknowns. We used the amino acid numbering of human PrP for reference, unless otherwise noted.

### Sample collection

Sample collection was focused on areas within and adjacent to the current CWD endemic area in Canada (using incidence data up to 2016; Figure 1). We obtained hair samples of bison (N = 48) from Parks Canada (collection permit EI-2017-25877), and DNA samples (N = 23) from archived samples [67]. Geographic information on these samples refers to their National Park of origin. Moose (N = 163) and elk (N = 27) samples in Alberta were collected through the Alberta Environment & Parks CWD surveillance programme, where hunters submit animal heads for testing, and a small portion of the ear is obtained for genetic analysis. Geographic information for most samples refers to the centroid of the Alberta township grid where the animal was harvested. Moose (N = 17), and elk (N = 18) samples from British Columbia were obtained through the Ministry of Environments surveillance programme; similar to Alberta, hunters submit animal heads for CWD testing, and a small tissue sample is obtained for genetic analysis. Geographic information for these samples refers to the centroid of the wildlife management region. Bighorn sheep tissue samples (N = 164) were archived samples (D. Coltman lab), and geographic data refers to nearest town or National Park centroid. Tissue samples from mountain goats (N = 95) were generously provided by A. Shafer. These samples were obtained from hunter harvested animals at compulsory inspection or registration stations (see [68]). Finally, pronghorn hair samples (N = 23) were obtained from Alberta Environment & Parks, their geographic information refers to wildlife management unit centroids.

### DNA extraction and Prnp sequencing

We extracted DNA from both hair and tissue samples using the DNeasy Blood & Tissue kit (Qiagen, Mississauga, ON) following the manufacturers protocol, eliminating the second elution step. Extracts were quantified using a NanoDrop 2000 (Thermo Fisher Scientific, Waltham, MA). We amplified the coding region of the *Prnp* gene in two fragments to meet the read-length requirements for next generation sequencing. We modified primers MD582F and MD1479R from Jewell et al [69]. and developed internal degenerate primers that would amplify across cervid and sympatric species (Table 2). These primers were tailed with transposase sequences to leave priming sites for the sequencing indices to be added. The chemical conditions for fragment 1 and 2 PCRs were as follows: 1 μL DNA, 1X 5X Q5 buffer with MgCl_2_, 2 mM dNTP, 10 uM overhang forward & reverse primers, 1X 5X High GC Enhancer, 5 U Q5 Hi-Fidelity DNA polymerase (New England Biolabs, Whitby, ON), and nuclease-free water in a total reaction volume of 10 μL. The cycling protocols for fragments 1 and 2 are included in Appendix 1.

**Table 2.** Primers used to amplify the *Prnp* coding sequence in two fragments across cervids and wild ungulates. The bold regions are the *Prnp* priming sites, and non-bold regions are the transposase sequences for adding indices for next-generation sequencing. The bold regions were used for Sanger sequencing.

Fragment	Sequence (5ʹ to 3ʹ)	Reference	
Fragment1_F	TCGTCGGCAGCGTCAGATGTGTATAAGAGACAG**ACRTGGGCATATGATGCTGAYACC**	[69]	MD582F
Fragment1_R	GTCTCGTGGGCTCGGAGATGTGTATAAGAGACAG**YTGCCAAAATGTATAAGAGG**		
Fragment2_F	TCGTCGGCAGCGTCAGATGTGTATAAGAGACAG**TGGAGGCTGGGGTCAAGG**		
Fragment2_R	GTCTCGTGGGCTCGGAGATGTGTATAAGAGACAG**ACTACAGGGCTGCAGGTAGAYACT**	[69]	MD1479R

To prepare the sequencing library for sequencing on the MiSeq platform (Illumina, San Diego, CA), we first completed quality/quantity assessment of the PCR fragments on agarose gels. We then combined the diluted fragments, from 1:10 to 1:100 depending on amplification success of species, at a 2:1 ratio of fragment one and fragment two, respectively. For indexing we used the Nextera XT (Illumina) indices purchased from IDT (Integrated DNA Technologies, Skokie, IL) as ultramers, with the following chemical conditions: 2 μL diluted, pooled fragments, 5X Q5 buffer with MgCl_2_, 2 mM dNTP, 5 μM index 1 (i7), 5 μM index 2 (i5), 5 U Q5 Hi-Fidelity DNA polymerase, and nuclease free water in a total reaction volume of 20 μL. The PCR cycling conditions were as follows: 95°C for 3 min, 8 cycles of 95°C for 30 s, 55°C for 30 s, 72°C for 30 s, followed by 72°C for 5 min. We used the NucleoMag NGS CleanUp (Macherey-Nagel, Düren, Germany) to purify our fragments for the sequencing library following the manufacturer’s protocol with these modifications: ratio of 0.75:1 (magnetic beads to pooled library), washed with 190 μL 80% ethanol, and eluted in 20 μL of nuclease-free water following ~10 min of drying. A small subset of samples was randomly selected at this point to assess quality and quantity of products on a Bioanalyzer (Agilent Technologies, Santa Clara, CA). Following the quality control, we quantified the library concentration on a Qubit^TM^ (Thermo Fisher Scientific), and diluted the library to 4 nM. Sequencing was completed by the Molecular Biology Service Unit at the University of Alberta, on a MiSeq using the Reagent V3 600 cycle reagent kit.

### Sequence assembly and polymorphism analysis

Sequence data were uploaded to BaseSpace (Illumina) as they were generated on the MiSeq. Following indexing of all samples, run data were downloaded and processed in Geneious R11 (Biomatters, Aukland, New Zealand). Once we set paired reads, we assembled each individual to cervid *Prnp* (Accession: AY228473 [69]), with the exception of bison and pronghorn, which were assembled using a bovine *Prnp* sequence (Accession: AY720448 [70]) as reference. The parameters we used were ‘Medium Sensitivity’, and each assembly was iterated up to 5 times, we completed trimming of the data on both 5ʹ and 3ʹ ends with a 0.05 error probability limit, and we saved a consensus sequence with a 65% threshold to ensure we were able to capture novel mutations.

We exported the consensus sequences as a fasta file, and sorted all genotypes using FaBox ver 1.4 [71]. Homozygous individuals were used to identify haplotypes in the populations, and we used this information to resolve the haplotypes among heterozygous individuals. Because there are very few mutations within species, identifying haplotypes was possible without knowing strand information. Individuals with novel mutations in the amino acid sequence not previously documented were Sanger sequenced in both the forward and reverse directions to confirm the mutation using the same PCR amplification protocol for the two fragments above using primers without the transposase overhangs (Table 2). Amplified fragments were cleaned for sequencing using ExoSAP-IT^TM^ (Thermo Fisher Scientific) following the manufacturer’s recommendations. Sequences were generated using the forward or reverse primer and the BigDye® Terminator v3.1 Cycle Sequencing Kit (Applied Biosystems, Foster City, CA), and data were generated on an ABI Prism 3730 DNA analyser (Applied Biosystems). Unique haplotypes were aligned to the sequence matrix of susceptible polymorphisms to compare amino acid composition at the sites associated with CWD susceptibility.

## Data accessibility

All alignments, and the reduced PrP matrix are available on the University of Alberta Dataverse repository (doi: https://doi.org/10.7939/DVN/Z5WSYW).
