# Supplementary material for: Predicting the spread-risk potential of chronic wasting disease to sympatric ungulate species
Source: Prion. 2020 Feb 3;14(1):56–66. doi: 10.1080/19336896.2020.1720486 (PMC7009333; doi:10.1080/19336896.2020.1720486)
Supplement: Supplemental Material [file kprn-14-01-1720486-s001.pdf]

Supplementary Information

Table S1 Amino acid accession and database sequence information for cytochrome b sequences used in trait evolution analyses including the resistance matrix for CWD (R = resistant, S = susceptible, U = unknown) for each species. For this analysis humans are used as the reference sequence and are assumed to be resistant to CWD infection.

Family	Species	CWD	Accession	Database source	RefSeq accession	Citation
Antilocapridae	*Antilocapra americana*	U	YP_007625795.1	RefSeq	NC_020679.1	Hassanin et al. 2012
Bovidae	*Bison bison*	U	YP_002791041.1	RefSeq	NC_012346.1	Achilli et al. 2008
Bovidae	*Bos taurus*	R	YP_209217.1	RefSeq	NC_006853.1	Chung and Ha 2005
Bovidae	*Oreamnos americanus*	U	YP_007625158.1	RefSeq	NC_020630.1	Hassanin et al. 2009
Bovidae	*Ovis canadensis*	U	YP_004769596.1	RefSeq	NC_015889.1	Miller et al. 2011
Canidae	*Canis latrans*	R	YP_626727.1	RefSeq	NC_008093.1	Björnerfeldt et al. 2006
Cebidae	*Saimiri sciureus*	S	YP_002929489.1	RefSeq	NC_012775.1	Matsui et al. 2009
Cercopithecidae	*Macaca fascicularis*	R	YP_002884238.1	RefSeq	NC_012670.1	Yi et al. 2009
Cervidae	*Alces alces*	S	YP_007625769.1	RefSeq	NC_020677.1	Hassanin et al. 2012
Cervidae	*Alces alces alces*	S	O47922.1	UniProt	NA	Randi et al. 1998
Cervidae	*Cervus canadensis canadensis*	S	AAL17840.1	Protein	NA	Ludt et al. 2004
Cervidae	*Muntiacus reevesi*	S	NP_663804.1	RefSeq	NC_004069.1	Zhang et al. 2010
Cervidae	*Odocoileus hemionus*	S	YP_007626445.1	RefSeq	NC_020729.1	Hassanin et al. 2012
Cervidae	*Odocoileus virginianus*	S	YP_004300520.1	RefSeq	NC_015247.1	Seabury et al. 2011
Cervidae	*Rangifer tarandus*	S	YP_448945.1	RefSeq	NC_007703.1	Wada et al. 2007
Cervidae	*Rangifer tarandus granti*	S	AAV84418.1	Protein	NA	Cronin et al. 2005
Cricetidae	*Phodopus sungorus*	R	ACG76170.1	Protein	NA	Meshchersky and Feoktistova 2009
Felidae	*Felis catus*	R	NP_008263.1	RefSeq	NC_001700.1	Lopez et al. 1996
Hominidae	*Homo sapiens*	R	YP_003024038.1	RefSeq	RefSeq	Anderson et al. 1981
Mustelidae	*Mustela putorius*	R	YP_007625262.1	RefSeq	NC_020638.1	Yu et al. 2011

Mustelidae	*Mustela putorius furo*	R	BAB08027.1	Protein	NA	Kurose et al. 2000
Mustelidae	*Neovison vison*	R	YP_007625301.1	RefSeq	NC_020641.1	Kurose et al. 2000
Procyonidae	*Procyon lotor*	R	YP_001096027.1	RefSeq	NC_009126.1	Takada et al. 2008

References

Achilli, Alessandro, Anna Olivieri, Marco Pellecchia, Cristina Uboldi, Licia Colli, Nadia Al-Zahery, Matteo Accetturo et al. "Mitochondrial genomes of extinct aurochs survive in domestic cattle." Current Biology 18, no. 4 (2008): R157-R158. https://doi.org/10.1016/j.cub.2008.01.019

Anderson, Sharon, Alan T. Bankier, Bart G. Barrell, Maarten HL de Bruijn, Alan R. Coulson, Jacques Drouin, Ian C. Eperon et al. "Sequence and organization of the human mitochondrial genome." Nature 290, no. 5806 (1981): 457. https://doi.org/10.1038/290457a0

Björnerfeldt, Susanne, Matthew T. Webster, and Carles Vilà. "Relaxation of selective constraint on dog mitochondrial DNA following domestication." Genome research 16, no. 8 (2006): 990-994. https://doi.org/10.1101/gr.5117706

Chung, H. Y., & Ha, J. M. (2005). Haplotype analysis of mitochondrial DNA in Korean native cattle. Unpublished, http://www. ncbi.nlm.nih.gov.

Cronin, Matthew A., Michael D. MacNeil, and John C. Patton. "Variation in mitochondrial DNA and microsatellite DNA in caribou (Rangifer tarandus) in North America." Journal of Mammalogy 86, no. 3 (2005): 495-505. https://doi.org/10.1644/1545-1542(2005)86[495:VIMDAM]2.0.CO;2

Hassanin, A., Delsuc, F., Ropiquet, A., Hammer, C., Van Vuuren, B.J., Matthee, C., Ruiz-Garcia, M., Catzeflis, F., Areskoug, V., Nguyen, T.T. and Couloux, A., 2012. Pattern and timing of diversification of Cetartiodactyla (Mammalia, Laurasiatheria), as revealed by a comprehensive analysis of mitochondrial genomes. Comptes Rendus Biologies, 335(1), pp.32-50. https://doi.org/10.1016/j.crvi.2011.11.002

Hassanin, Alexandre, Anne Ropiquet, Arnaud Couloux, and Corinne Cruaud. "Evolution of the mitochondrial genome in mammals living at high altitude: new insights from a study of the tribe Caprini (Bovidae, Antilopinae)." Journal of molecular evolution 68, no. 4 (2009): 293-310. https://doi.org/10.1007/s00239-009-9208-7

Kurose, Naoko, Alexei V. Abramov, and Ryuichi Masuda. "Intrageneric diversity of the cytochrome b gene and phylogeny of Eurasian species of the genus Mustela (Mustelidae, Carnivora)." Zoological Science 17, no. 5 (2000): 673-680. https://doi.org/10.2108/zsj.17.673

Lopez, Jose V., Stanley Cevario, and Stephen J. O'Brien. "Complete nucleotide sequences of the domestic cat (Felis catus) mitochondrial genome and a transposed mtDNA tandem repeat (Numt) in the nuclear genome." Genomics 33, no. 2 (1996): 229-246. https://doi.org/10.1006/geno.1996.0188

Ludt, Christian J., Wolf Schroeder, Oswald Rottmann, and Ralph Kuehn. "Mitochondrial DNA phylogeography of red deer (Cervus elaphus)." Molecular phylogenetics and evolution 31, no. 3 (2004): 1064-1083. https://doi.org/10.1016/j.ympev.2003.10.003

Matsui, Atsushi, Felix Rakotondraparany, Isao Munechika, Masami Hasegawa, and Satoshi Horai. "Molecular phylogeny and evolution of prosimians based on complete sequences of mitochondrial DNAs." Gene 441, no. 1-2 (2009): 53-66. https://doi.org/10.1016/j.gene.2008.08.024

Meshchersky, I. G., and N. Yu Feoktistova. "Intraspecific organization of dwarf hamsters Phodopus campbelli and Phodopus sungorus (Rodentia: Cricetinae) basing on mtDNA analysis." In Doklady Biological Sciences, vol. 424, no. 1, pp. 35-38. MAIK Nauka/Interperiodica, 2009. https://doi.org/10.1134/S0012496609010116

Miller, Joshua M., René M. Malenfant, Stephen S. Moore, and David W. Coltman. "Short reads, circular genome: skimming solid sequence to construct the bighorn sheep mitochondrial genome." Journal of Heredity 103, no. 1 (2011): 140-146. https://doi.org/10.1093/jhered/esr104

Randi, Ettore, Nadia Mucci, Massimo Pierpaoli, and Emmanuel Douzery. "New phylogenetic perspectives on the Cervidae (Artiodactyla) are provided by the mitochondrial cytochrome b gene." Proceedings of the Royal Society of London. Series B: Biological Sciences 265, no. 1398 (1998): 793-801. https://doi.org/10.1098/rspb.1998.0362

Seabury, Christopher M., Eric K. Bhattarai, Jeremy F. Taylor, Ganesh G. Viswanathan, Susan M. Cooper, Donald S. Davis, Scot E. Dowd, Mitch L. Lockwood, and Paul M. Seabury. "Genome-wide polymorphism and comparative analyses in the white-tailed deer (Odocoileus virginianus): a model

for conservation genomics." PLoS One 6, no. 1 (2011): e15811. https://doi.org/10.1371/journal.pone.0015811

Takada,Y., Mukaida,M. and Imai,T. 2008. Unpublished, http://www. ncbi.nlm.nih.gov.

Wada, Kenta, Masahide Nishibori, and Michinari Yokohama. "The complete nucleotide sequence of mitochondrial genome in the Japanese Sika deer (Cervus nippon), and a phylogenetic analysis between Cervidae and Bovidae." Small ruminant research 69, no. 1-3 (2007): 46-54. https://doi.org/10.1016/j.smallrumres.2005.12.002

Yi,D., Guo,Y., Jiang,Q., Fan,J. and Lan,G. (2009). Sequencing and analysis of the complete mitochondrial genome of the *Macaca fascicularis*. Unpublished, http://www. ncbi.nlm.nih.gov.

Yu, Li, Dan Peng, Jiang Liu, Pengtao Luan, Lu Liang, Hang Lee, Muyeong Lee, Oliver A. Ryder, and Yaping Zhang. "On the phylogeny of Mustelidae subfamilies: analysis of seventeen nuclear non-coding loci and mitochondrial complete genomes." BMC Evolutionary Biology 11, no. 1 (2011): 92. https://doi.org/10.1186/1471-2148-11-92

Zhang,X.M., Zhang,H.J., Mou,Y., Li,J., Yi,G.C. and Shan,X.N. 2010. Unpublished, http://www. ncbi.nlm.nih.gov.

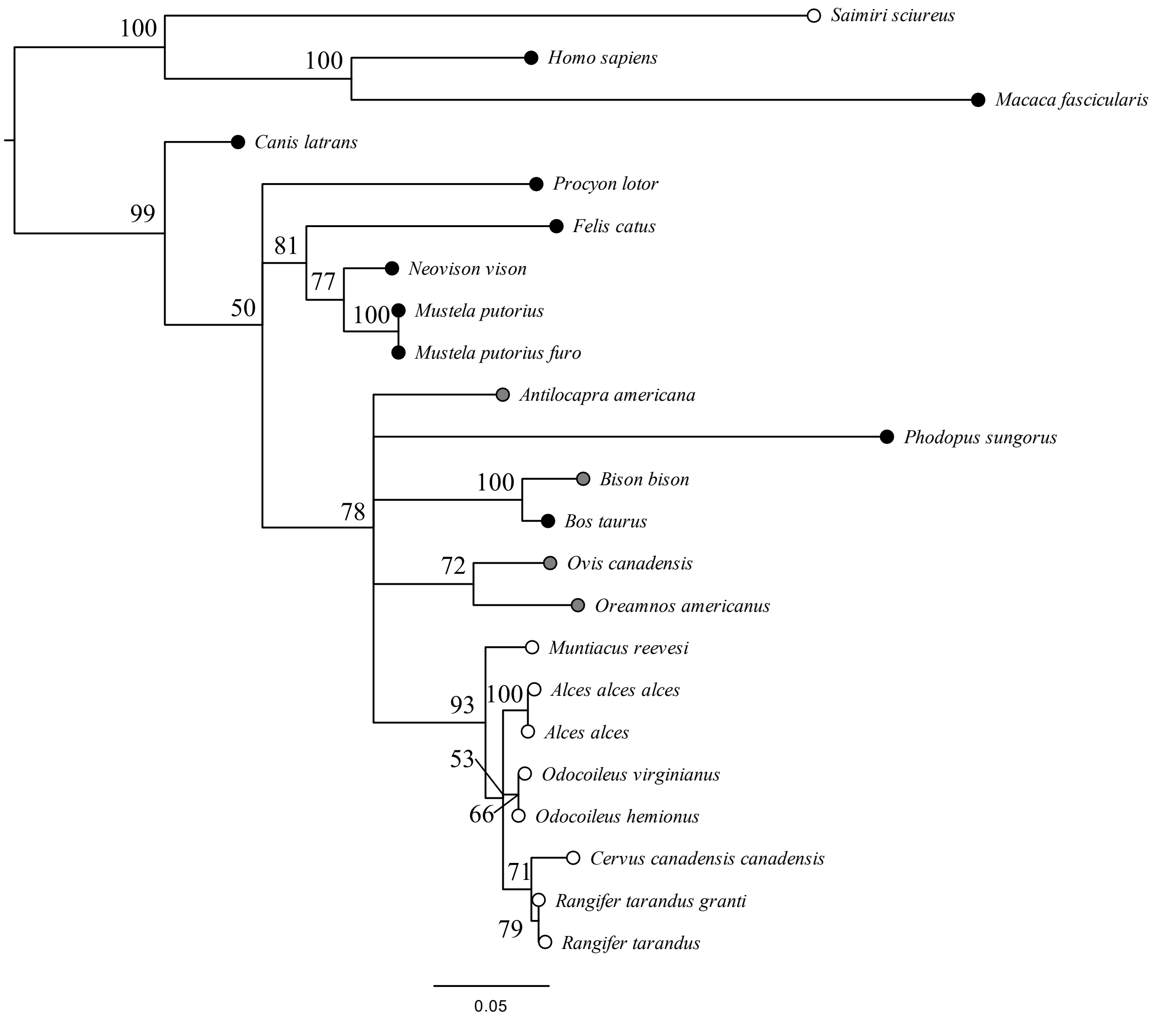

Figure S1. Maximum likelihood phylogram of cytochrome b used in ancestral and unknown state predictions. Branches with less than 50% bootstrap support were collapsed. Susceptible species indicated with white circles at branch tips, resistant species with black circles, and unknown with grey.

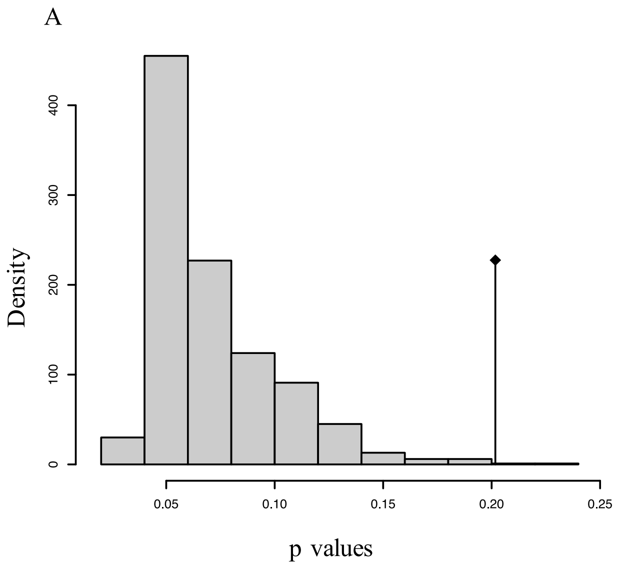

Figure S2. Susceptibility to CWD and phylogenetic signal are correlated with (A; p = 0.003) and without branch lengths (B; p = 0.001) as a factor. Significance of correlation tested using Moran's I with relationships among taxa inferred from the 19 species cytochrome b phylogeny (Fig. 2A) that does not have any missing infection data.

Additional File 1 - scripts

Model testing:

```
phyml -i infile.phy -d aa -m MTMAM -f m -v e -a e -c 4 -o tlr

raxmlHPC-SSE3 -s infile.phy -m PROTGAMMAIMTMAM -n EXEC_NAME -p PARSIMONY_SEED

raxml-ng --msa infile.phy --model MTMAM+I+G4

paup -s infile.phy

iqtree -s infile.phy -m MTMAM+I+G4
```

Running raxml:

```
raxmlHPC-HYBRID -T 4 -s infile -N autoMRE -n result -k -f a -p 12345 -x 12345 -m PROTCATMTMAMF
```

Adding BS values to raxml best tree:

```
raxmlHPC-AVX -f b -t RAxML_bestTree.result -z RAxML_bootstrap.result -m PROTCATMTMAMF -n BStree
```

Phylogenetic correlation

```
setwd("~/Documents/sidePieces/CWD/prnpsequencedata")

library(adephylo)

library(ape)

library(phylobase)

prnpTre <- read.tree("RAxML_bipartitions.BStree")

plot(prnpTre)

suscpetMatr <- read.table("SusceptMatr.txt", header = T, sep = "\t",as.is = 1)

suscpetMatr <- data.frame(suscpetMatr, row.names = 1)

prnpTre$node.label<-c()

prnpData <- phylo4d(prnpTre, suscpetMatr)

table.phylo4d(prnpData)

prnp.abTest <- abouheif.moran(prnpData)

prnp.abTest

plot(prnp.abTest)

prnp.prox <- vcv.phylo(prnpTre)

prnp.abTest2 <- abouheif.moran(prnpData, W=prnp.prox)

prnp.abTest2
```
